# Case report: Personalized, response-adapted neoadjuvant alectinib achieves durable remission in stage IIIB ALK-rearranged lung cancer

**DOI:** 10.3389/fphar.2026.1758062

**Published:** 2026-05-19

**Authors:** Hui Shen, Sishi Huang, Jianbin Zhang

**Affiliations:** 1 Department of Respiratory Medicine, Huzhou Central Hospital, Fifth School of Clinical Medicine of Zhejiang Chinese Medical University, Huzhou, Zhejiang, China; 2 Department of Thoracic Surgery, Huzhou Central Hospital, Affiliated Central Hospital of Huzhou University, Huzhou, Zhejiang, China

**Keywords:** alectinib, case report, neoadjuvant therapy, non-small cell lung cancer, response-adapted strategy

## Abstract

**Background:**

Alectinib, a second-generation ALK inhibitor, is established as a first-line treatment for advanced ALK-positive NSCLC and as adjuvant therapy following resection for early-stage disease. However, its role as a neoadjuvant therapy remains under investigation.

**Case presentation:**

We report a 57-year-old never-smoking woman diagnosed with stage IIIB (cT4N2M0) ALK-rearranged lung adenocarcinoma. After the initial multidisciplinary team (MDT) convening, a consensus was reached to initiate neoadjuvant alectinib (600 mg twice daily), with the first response evaluation scheduled at 4 weeks. The first restaging computed tomography (CT) demonstrated a significant partial response (PR), characterized by a 76% reduction in the primary tumor diameter and a 50% reduction in the short axis of the station 7 lymph node. In light of this robust response which already met surgical criteria, the MDT decided to proceed with a second 4-week consolidation cycle to maximize the pathological response prior to surgery. Following the second cycle, subsequent imaging confirmed further tumor regression. The patient then underwent successful R0 resection, with pathology confirming a major pathological response (MPR). Adjuvant alectinib was resumed on postoperative day 2. At the last follow-up (33 months post-surgery), the patient remains disease-free with no clinically significant treatment-related adverse events.

**Conclusion:**

This case illustrates the successful personalization of neoadjuvant alectinib by employing an imaging response-adapted strategy. This strategy utilized dynamic imaging assessments to guide the scheduling of the surgical procedure, culminating in a deep pathological response and prolonged disease-free survival, thereby offering a refined perioperative paradigm for ALK-rearranged NSCLC.

## Introduction

The management of locally advanced non-small cell lung cancer (NSCLC) has been increasingly focused on the development of effective neoadjuvant strategies to improve surgical outcomes and long-term survival (Provencio et al., 2023). Neoadjuvant immunotherapy has been established as a standard treatment (Forde et al., 2022). However, its efficacy is substantially limited in oncogene-driven subsets, such as anaplastic lymphoma kinase (ALK)-rearranged tumors, which show intrinsic resistance to immune checkpoint inhibition and higher immune-related toxicity (Gainor et al., 2016). While platinum-based chemotherapy remains an effective neoadjuvant option, its overall survival benefit in this specific population is modest (NSCLC Meta-analysis Collaborative Group, 2014). These collective limitations of conventional modalities have therefore propelled the investigation of targeted therapies as a rational and promising approach in the neoadjuvant setting, aiming to leverage their high efficacy and favorable tolerability (Liu et al., 2022).

ALK-tyrosine kinase inhibitors (TKIs), with their established efficacy and favorable safety profile in ALK-rearranged NSCLC, represent a promising neoadjuvant strategy (Kwak et al., 2010). Alectinib, a second-generation ALK-TKI, has been proven effective and safe in metastatic (Camidge et al., 2019) and adjuvant (Wu et al., 2024) settings. This proven profile has prompted its evaluation in the neoadjuvant setting. The phase II ALNEO trial (Leonetti et al., 2021) was the first prospective study designed specifically to evaluate alectinib in the neoadjuvant setting for resectable stage III ALK-positive NSCLC, reporting feasibility and major pathological response in its initial case. These promising findings are further supported by several case reports (Shi et al., 2023; Sentana-Lledo et al., 2022) which also confirm the feasibility and profound pathological efficacy of this approach. Nevertheless, a framework for personalizing neoadjuvant alectinib treatment is still lacking, posing a risk of either undertreatment, which may compromise pathological outcomes, or overtreatment, leading to unnecessary drug exposure and surgical delays. Moreover, data on long-term survival remain scarce.

Herein, we describe the first detailed application of a dynamic, imaging response-adapted strategy for neoadjuvant alectinib in a patient with stage IIIB ALK-rearranged NSCLC. This strategy facilitated R0 resection, a major pathological response (MPR), and sustained disease-free survival.

## Case presentation

### Baseline evaluation and diagnosis

**FIGURE 1:**
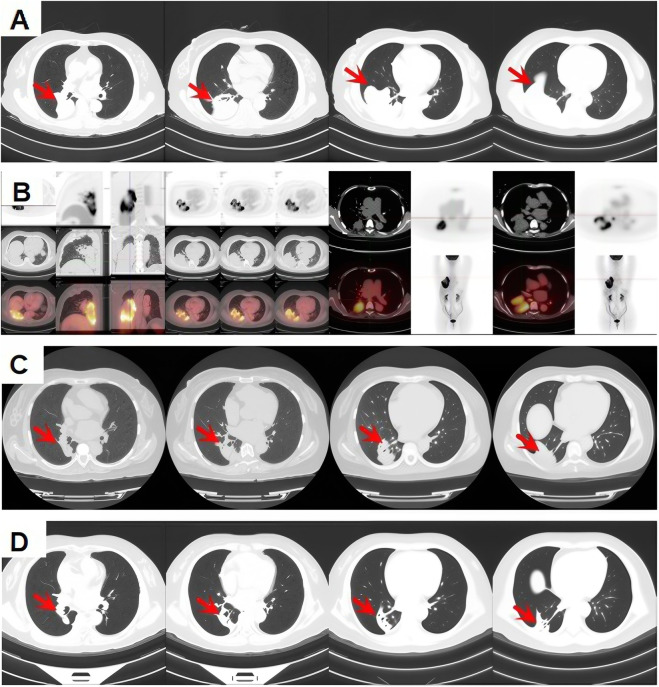
Radiologic Assessments Before and After Neoadjuvant Alectinib. **(A)** Baseline contrast-enhanced chest CT at diagnosis shows a large lesion (8.3 cm) in the right lower lobe (arrows). **(B)** Baseline positron emission tomography-computed tomography (PET-CT) image demonstrates intense FDG avidity in the primary tumor (SUVmax 17.59) and station 11R/7 lymph nodes (SUVmax 4.99). **(C)** CT scan after 4 weeks of alectinib treatment shows a marked partial response, with significant regression of both the primary tumor (arrows) and nodal disease. **(D)** CT scan after 8 weeks of alectinib therapy demonstrates continued tumor regression (arrows) and consolidation of the treatment response.

**FIGURE 2:**
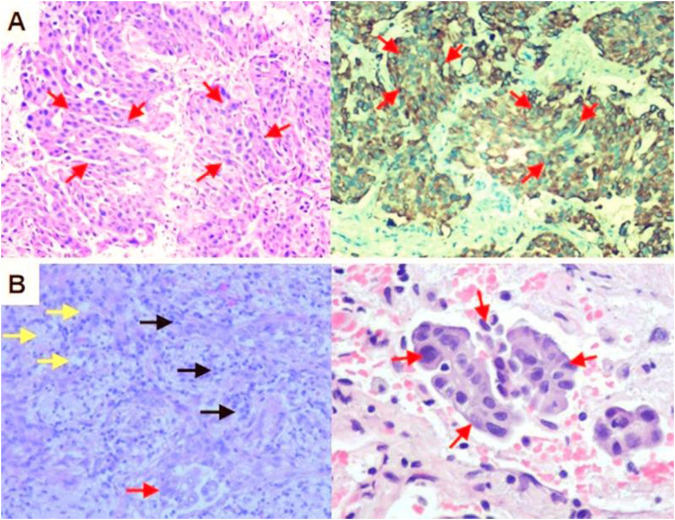
Histopathologic Correlates of Treatment Response. **(A)** Hematoxylin and eosin (H&E) staining of the transbronchial lung biopsy specimen obtained at diagnosis confirms poorly differentiated solid adenocarcinoma (arrows). **(B)** H&E staining of the resected primary tumor specimen following 8 weeks of neoadjuvant alectinib reveals a major pathological response (MPR). Residual tumor cells (red arrows), foam cells (yellow arrows), and inflammatory infiltrates (black arrows) are highlighted.

A 57-year-old never-smoking woman presented in October 2022 with a 4-month history of persistent cough. She had no significant personal or family history of cancer. Physical examination was unremarkable. Contrast-enhanced CT at our center confirmed a central mass measuring 8.9 cm × 10.6 cm with right hilar and mediastinal lymphadenopathy, suggesting malignancy (Figure 1A). Positron emission tomography-computed tomography (PET-CT) revealed high fluorodeoxyglucose (FDG) uptake in the primary lesion (SUVmax = 17.59) and lymph nodes at stations 11R and 7 (SUVmax = 4.99) (Figure 1B). Serum carcinoembryonic antigen (CEA) was elevated at 82.22 ng/mL (normal: 0–5.00 ng/mL). Fiberoptic bronchoscopy showed hyperemic and edematous mucosa with luminal narrowing in the right lower lobe. Histopathology of specimens from transbronchial lung biopsy and needle aspiration of stations 11R and 7 confirmed poorly differentiated solid adenocarcinoma (Figure 2A). Molecular profiling by amplification refractory mutation system polymerase chain reaction (ARMS-PCR) identified an ALK rearrangement. The patient was diagnosed with stage IIIB (cT4N2M0) ALK-rearranged lung adenocarcinoma (TNM Classification, 8th edition).

### Implementation of the response-adapted strategy

Upon diagnosing stage IIIB ALK-rearranged lung adenocarcinoma, the multidisciplinary team (MDT) decided to employ neoadjuvant alectinib, adopting a personalized response-adapted strategy instead of a fixed-duration model. The cornerstone of this approach was the use of early imaging assessments to dynamically steer therapeutic decisions, with the first response-evaluation CT scheduled at 4 weeks. The 4-week CT scan results (Figure 1C) confirmed the strategy’s validity, demonstrating a partial response (PR) with a 76% reduction in the primary tumor diameter and a 50% reduction in the short axis of the station 7 lymph node. This significant tumor regression met the radiological criteria for R0 resection. Nevertheless, the MDT opted to extend neoadjuvant therapy for an additional 4 weeks. This decision was driven by two complementary goals: to deepen the pathological response, and more critically, to avoid a combined middle lobectomy, thereby achieving R0 resection while maximizing pulmonary function preservation. The patient provided informed consent and received this additional therapy cycle.

### Surgical intervention and pathological evaluation

After two 4-week cycles of neoadjuvant alectinib (600 mg twice daily), repeat CT confirmed further significant regression (Figure 1D). Following final MDT approval, the patient underwent video-assisted thoracoscopic surgery (VATS) right lower lobectomy with systematic mediastinal lymph node dissection on 30 December 2022. Operative time was 127 min, estimated blood loss was 50 mL, and no intraoperative complications were encountered. Final pathology confirmed an MPR, with only about 5% residual viable tumor cells (Figure 2B). All dissected lymph nodes were tumor-free, with no vascular or lymphatic invasion.

### Postoperative course and follow-up

**FIGURE 3:**
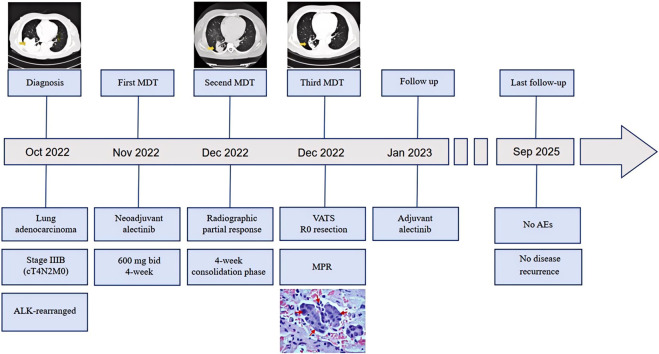
Clinical Timeline. Schematic illustration of the patient’s management course, from diagnosis of stage IIIB ALK-rearranged adenocarcinoma through neoadjuvant alectinib, response-adapted treatment extension, R0 resection with major pathological response (MPR), adjuvant alectinib, and sustained disease-free survival (DFS) of 33 months.

The patient recovered well after surgery. Adjuvant alectinib was resumed at the same dose (600 mg twice daily) on postoperative day 2, as planned, and was well-tolerated. At the last follow-up in September 2025 (33 months post-surgery), the patient remains disease-free and continues on adjuvant alectinib, with a planned total duration of 36 months. The clinical course is summarized in Figure 3.

## Discussion

This report delineates the successful implementation of a dynamic, imaging response-adapted strategy to personalize neoadjuvant alectinib, culminating in a major pathological response (MPR), successful R0 resection, and sustained disease-free survival (DFS) exceeding 33 months in a patient with stage IIIB ALK-rearranged NSCLC. This case provides a compelling paradigm for optimizing the perioperative management of locally advanced ALK-rearranged NSCLC.

Anaplastic lymphoma kinase (ALK) rearrangement is a well-established oncogenic driver, occurring in approximately 3%–7% of NSCLC patients (Kwak et al., 2010). The profound sensitivity of ALK-rearranged tumors to ALK inhibition stems from their “oncogene addiction,” a pharmacological concept where cancer cell survival becomes dependent on a single oncogenic pathway (Soda et al., 2008). This exquisite dependency renders them highly vulnerable to targeted inhibition, earning this subtype the distinction of a “diamond mutation.” Alectinib, a second-generation ALK-TKI, exemplifies this principle. It possesses high potency and selectivity for ALK, and is engineered to overcome the resistance mutations and poor central nervous system penetration associated with earlier-generation inhibitors (Camidge et al., 2019; Ou et al., 2016). Its established efficacy and favorable safety profile have cemented its role as a first-line standard for advanced disease and adjuvant therapy following resection (Camidge et al., 2019; Wu et al., 2024). Capitalizing on this robust pharmacological foundation, the exploration of neoadjuvant alectinib for locally advanced ALK-positive NSCLC has garnered significant interest. Emerging evidence has consistently confirmed the feasibility and profound pathological efficacy of this approach. The phase II ALNEO trial (NCT03858478) (Leonetti et al., 2021), a pivotal open-label, single-arm, multicenter study, was designed to prospectively evaluate alectinib as neoadjuvant therapy in patients with resectable stage III ALK-positive NSCLC. This landmark study established the proof-of-concept for this approach, with its protocol defining the achievement of a major pathological response (MPR) as the primary endpoint. This is further corroborated by several case reports detailing pCR. For instance, Shi et al. (Shi et al., 2023) reported two patients with stage IIIB disease achieving pCR after an extended neoadjuvant course of approximately 15.6 weeks, while Wang et al. (2023) in their case series and literature review reported two patients with stage IIIB disease who achieved pCR after neoadjuvant alectinib.

Despite these encouraging results, a significant challenge persists: the absence of a personalized framework for neoadjuvant alectinib, which creates uncertainty in two critical, interrelated domains--the optimal treatment duration and the optimal timing for surgical intervention. As summarized in Table 1, the current landscape is dominated by fixed-duration regimens, which exhibit considerable heterogeneity and carry inherent risks due to their inflexibility. For instance, a fixed 6-week course in one report led to a suboptimal pathological response (non-MPR) and only 12 months of disease-free survival (DFS), suggesting potential undertreatment and premature surgery (Yue et al., 2021). Conversely, one study documented the achievement of pathological complete response (pCR) following an extended fixed duration of alectinib exceeding 30 weeks (Shi et al., 2023). While effective, such a protracted regimen for a rapidly responding tumor may represent overtreatment and an unnecessary delay of curative-intent surgery. This “one-size-fits-all” paradigm, which fails to individualize both the therapeutic exposure and the surgical timeline, underscores the critical need for a more dynamic approach. Although some prior studies incorporated early imaging evaluations (Leonetti et al., 2021; Yue et al., 2021), these assessments did not translate into dynamic therapeutic or surgical adjustments, missing crucial opportunities for personalization.

**TABLE 1:** Summary of reported cases on neoadjuvant alectinib in ALK-rearranged NSCL**C**.

Study (first author, year)	Study type	Number of patients	Disease stage	Time to first radiologic evaluation	Neoadjuvant therapy duration	Pathological outcome	Adjuvant therapy	DFS (M)
Leonetti et al. (2021)	Phase II Trial (ALNEO)	Multiple (Trial)	III (Resectable)	Not specified	Not specified	pCR/MPR reported	Not specified	Ongoing
Yue et al. (2021)	Case Report	1	IIIA	Post-treatment	6 weeks	Non-MPR	Yes	12
Hu et al. (2022)	Case Report	1	IIIA	Post-treatment	8 weeks	pCR	Not specified	Not reported
Sentana-Lledo et al. (2022)	Case Report	1	IIIA (N2)	Post-treatment	12 weeks	pCR	No	12
Wang et al. (2023)	Case Series	2	IIIB	Post-treatment	∼6.3 weeks (44 days)	pCR	Yes	24
Shi et al. (2023)	Case Report	2	IIIA/IIIB	Post-treatment	∼15.6 weeks (109 days)	pCR (2/2)	Yes	18
Cao et al. (2023)	Case Report	1	Locally Advanced	Post-treatment	Not specified	Inconsistent response	Not specified	Not reported
Xue et al. (2025)	Case Report	1	IIIB	Post-treatment	8 weeks	pCR	Yes	26
Current Case, 2025	Case Report	1	IIIB	On-treatment (4 weeks)	(4 + 4) weeks (response-adapted)	MPR	Yes	33

MPR, major pathological response (≤10% residual viable tumor); pCR, pathological complete response; DFS, disease-free survival; M, months.

Our case directly addresses this dual challenge by introducing a novel, imaging response-adapted strategy. The pivotal innovation lies in utilizing the 4-week restaging CT as a definitive decision point within a dynamic “assess-and-adapt” framework. In contrast to the fixed regimens that predefine both treatment length and surgery date, the observation of a significant partial response (PR) at 4 weeks directly informed our MDT’s decision to administer a single consolidation cycle. This approach dynamically tailored the total drug exposure (8 weeks) and correspondingly refined the timing for surgery, culminating in an MPR. This strategy fundamentally distinguishes itself by dynamically aligning both treatment intensity and surgical planning with individual tumor response, thereby optimizing the entire perioperative therapeutic window.

The robust long-term outcome in our patient (33 months of DFS in stage IIIB disease) compares well with existing data (Table 1) and begins to address the scarcity of long-term survival evidence in this context. This sustained remission, surpassing the DFS reported in several other cases (Sentana-Lledo et al., 2022; Hu et al., 2022; Xue et al., 2025; Cao et al., 2023), likely reflects the synergistic effect of a comprehensive perioperative strategy. This triad integrates a response-adapted neoadjuvant phase to achieve deep tumor regression, followed by R0 surgical resection, and consolidated with adjuvant TKI therapy. The critical importance of this integrated approach is underscored by reports where the omission of adjuvant TKI, even after achieving pCR, resulted in early disease recurrence (Sentana-Lledo et al., 2022).

The role of postoperative ALK-TKI therapy in consolidating long-term disease control warrants emphasis. In our patient, early resumption of adjuvant alectinib on postoperative day 2 appeared to be associated with the sustained 33-month DFS. Recent reports have shown that even after deep pathological responses, omission of postoperative ALK-TKI may result in early distant recurrence, including brain metastases (Aoki et al., 2024; Ohta et al., 2025). Furthermore, the phase III ALINA trial (NCT03456076) established a significant disease-free survival benefit with adjuvant alectinib in resected ALK-positive NSCLC, supporting 3 years of postoperative therapy (Wu et al., 2024). Collectively, these findings underscore the importance of an integrated perioperative strategy—response-adapted neoadjuvant therapy, R0 resection, and adjuvant alectinib—as a comprehensive approach to optimizing outcomes in this patient population.

We acknowledge the inherent limitations of a single-case report. While our findings are promising, they require validation in larger, prospective cohorts to establish the generalizability of this response-adapted protocol. Furthermore, our assessment relied primarily on radiological and pathological evaluations. The incorporation of serial biomarker analyses, such as circulating tumor DNA (ctDNA) monitoring, in future iterations of this strategy could provide a more granular, molecular-level assessment of treatment response and further refine personalization.

In conclusion, we report the first successful case employing an imaging response-adapted strategy to personalize neoadjuvant alectinib therapy. This approach proved both feasible and highly effective. By dynamically tailoring treatment duration to radiological response, it refines current perioperative management and shows great promise for optimizing outcomes in patients with resectable ALK-rearranged NSCLC.

## Data Availability

The original contributions presented in the study are included in the article/supplementary material, further inquiries can be directed to the corresponding author.
